# The clinical characteristics and prognosis in adult Ph negative acute lymphoblastic leukemia with TP53 aberrations

**DOI:** 10.1186/s40164-022-00274-1

**Published:** 2022-04-08

**Authors:** Qiuyun Fang, Xiaoyuan Gong, Kaiqi Liu, Yujiao Jia, Yang Song, Guangji Zhang, Yan Li, Qishan Hao, Yueshen Ma, Shuning Wei, Bingcheng Liu, Ying Wang, Hui Wei, Jianxiang Wang, Yingchang Mi

**Affiliations:** State Key Laboratory of Experimental Hematology, National Clinical Research Center for Blood Diseases, Haihe Laboratory of Cell Ecosystem, Institute of Hematology & Blood Diseases Hospital, Chinese Academy of Medical Sciences & Peking Union Medical College, Tianjin, 300020 China

**Keywords:** Clinical characteristics, Prognosis, *TP53* aberrations, Ph negative acute lymphoblastic leukemia

## Abstract

Very few reports elucidate the prognosis of patients with *TP53* aberrations using both measurable residual disease (MRD) and the status of having undergone allogeneic hematopoietic stem cell transplantation (allo-SCT). In this study, aberrations of *TP53* were analyzed using next-generation sequencing (NGS) and fluorescence in situ hybridization (FISH) in patients with Philadelphia chromosome-negative (Ph^−^) ALL enrolled in a prospective single-arm clinical trial at our leukemia center. We analyzed the survival of the patients grouped according to the MRD level at the third month and whether or not received allo-SCT. We found that allo-SCT could improve the OS in patients with *TP53* aberrations; Patients having negative MRD at the third month still showed worse 3-year OS and 3-year DFS without undergoing allo-SCT, which is different from previous studies, moreover, the prognostic significance of *TP53* deletions was as important as *TP53* mutations, the importance of screening both *TP53* deletions and mutations in adult Ph^−^ ALL at diagnosis should be emphasized.

**Supplementary Information:**

The online version contains supplementary material available at 10.1186/s40164-022-00274-1.

## To the Editor,

*TP53* aberrations are one of the most common genetic lesions associated with cancers in humans, particularly with hematological malignancies [1]. Examples of genetic modifications include mutations, deletions, and insertions. Previous studies have found that the frequency of *TP53* aberrations observed in acute lymphoblastic leukemia (ALL) was 16–19%, which is higher than that observed in acute myeloid leukemia and myelodysplastic syndrome [2, 3]. *TP53* aberrations were strongly correlated with complex karyotype in ALL (45% of patients with complex karyotypes) and older age (25–36% of patients ≥ 60 years with *TP53* aberrations) [2]. Moreover, *TP53* aberrations resulted in suboptimal treatment response and poor survival rates [lower event-free survival and overall survival (OS) rates] in pediatric and adult patients with ALL [4–6]. Very few reports elucidate the prognosis of patients with *TP53* aberrations using both measurable residual disease (MRD) and the status of having undergone allogeneic hematopoietic stem cell transplantation (allo-SCT).

**Fig. 1:**
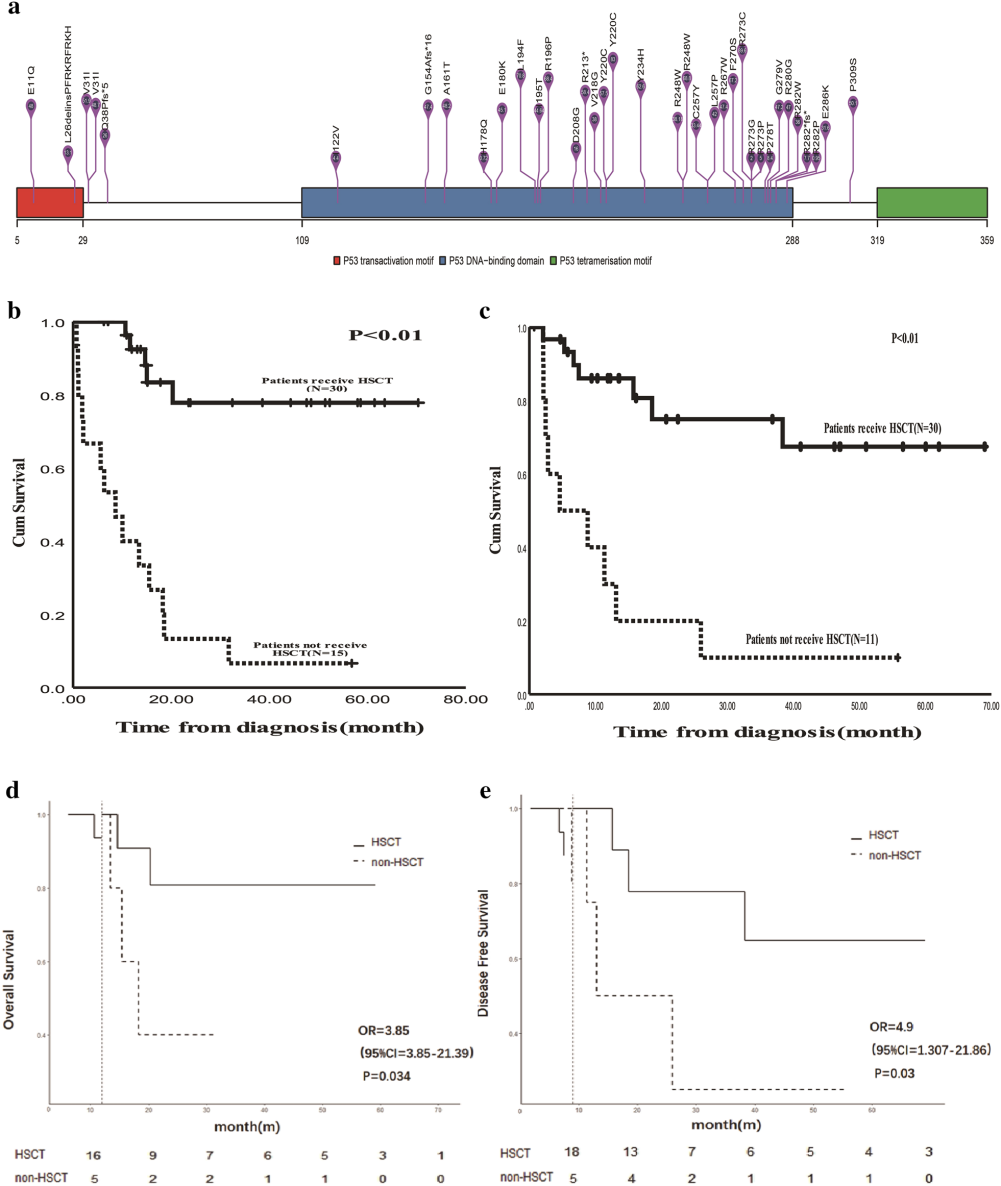
**A** The specific *TP53* gene mutation site from the 31 patients. **B**, **C** The 3-year OS and 3-year DFS of the 45 patients with the *TP53* aberrations. **D**, **E** The landmark analysis of the 3-year OS and 3-year DFS in the MRD negative ALL patients with *TP53* aberrations who underwent allo-SCT vs. the patients who did not undergo allo-SCT

Aberrations of *TP53* were analyzed using next-generation sequencing (NGS) (*n* = 309) and fluorescence in situ hybridization (FISH) (*n* = 242) in 309 patients with Philadelphia chromosome-negative (Ph^−^) ALL enrolled in a prospective single-arm clinical trial (ChiCTR-TNC-09000397) at our leukemia center [7]. The results revealed that 45 patients (14.6%; 25 men and 20 women) had *TP53* aberrations, which was lower than the proportions of patients reported in previous data. Of the 45 patients with *TP53* aberrations, 35 cases were B-cell ALL and 10 cases were T-cell ALL. The median patient age was 27 years (range: 14–67 years), and the median white blood cell count at diagnosis was 5.35 × 10^9^/L (range: 0.73–245 × 10^9^/L). Among the 45 patients, fourteen patients (14/242, 5.8%) had only *TP53* deletions (as per FISH), nine patients (9/242, 3.7%) had both *TP53* deletions and mutations, and 22 patients (22/309, 7.1%) had only *TP53* mutations (as per NGS). Detailed data of the patients with different *TP53* aberrations are listed in Table 1 (the concomitant gene mutations observed in the patients with *TP53* aberrations are listed in Additional file 1: Table S1). No significant differences were observed between the groups in terms of clinical characteristics. Median mutation frequency of *TP53* was 45.1% (range: 2–89.6%). Mutation sites were mainly located in exons 3–9 and included hotspot residues of R280, R273, R282, E286, C257, R248, Y220, R196, R213, and L194. Specific mutation sites are shown in Fig. 1A.

**Table 1:** The clinical characteristics of the 45 patients

	Total (N = 45)	TP53 del (N = 14)	TP53 mut (N = 22)	TP53 del & mut (N = 9)
Gender				
Male	25 (55.6%)	9 (64.3%)	10 (45.5%)	6 (66.7%)
Female	20 (44.4%)	5 (35.7%)	12 (54.5%)	3 (33.3%)
Age	27 (14–67)	21(14–57)	32 (14–53)	26 (14–59)
Diagnosis				
B-ALL	35 (77.8%)	9 (64.3%)	17 (77.3%)	9 (100%)
T-ALL	10 (22.2%)	5 (35.7%)	5 (22.7%)	0
WBC count	5.35 (0.73–245)	3.06 (0.73–245)	8.3 (1.03–100.77)	4.73 (1.21–47.6)
Cytogenetic stratification^a^				
Standard risk	35 (77.8%)	14 (100%)	16 (72.7%)	5 (55.6%)
High risk	10 (22.2%)	0	6 (27.3%)	4 (44.4%)
CR^b^	41 (100%)	12(100%)	20 (100%)	9 (100%)
MRD (3rd month)^c^				
Negative	23 (67.6%)	7 (87.5)	11 (64.7%)	5 (55.6%)
Positive	11 (32.4%)	1 (12.5%)	6 (35.3%)	4 (44.4%)
HSCT (41 CR patients)				
Yes	30 (73.2%)	9 (75%)	14 (70%)	7 (77.8%)
No	11 (26.8%)	3 (25%)	6 (30%)	2 (22.2%)

^a^The cytogenetic stratification is referred to NCCN guideline (Version 3.2021)

^b^The therapeutic effect could be evaluated in 41 patients, including 12 patients with TP53 deletion, 20 patients with TP53 mutation, and 9 patients with both TP53 deletion and mutation (del & mut)

^c^Thirty-four in the 45 patients had the MRD results on the third month from the beginning of therapy

All patients received scheduled therapy in accordance with the trial protocol described previously [7]. Three patients died during induction therapy, and one patient was lost during follow-up. The complete remission (CR) rate achieved after one course of induction therapy was 85.4% (35/41), and the overall CR rate was 100% (41/41). Eight-color flow cytometry performed during the third month after induction therapy initiation revealed the MRD level of 35 patients, of whom 23 (65.5%) exhibited negative results (MRD level < 0.01%) and 11 (34.5%) exhibited positive results. Of the 41 patients who achieved CR, 30 patients underwent allo-SCT during the first CR period (CR1) and 11 did not undergo allo-SCT.

We analyzed the data from several different perspectives (Additional file 1: Data S1, Tables S2, S3, Fig. S1) and confirmed that *TP53* aberration is a poor independent prognostic factor for Ph^−^ ALL. The median follow-up time was 38.57 months (range: 17.77–51.35 months). The 3-year OS rate of the 45 patients was 49.4% ± 8.6%, and the 3-year disease-free survival (DFS) rate was 50.7% ± 9.6%. For patients with different *TP53* aberrations, the 3-year OS and 3-year DFS rates showed no significant difference (deletion only vs. mutation only vs. deletion and mutation: 3-year OS: 48.9% ± 15.6% vs. 53% ± 12% vs. 40% ± 20.3%, *p* = 0.948; 3-year DFS: 47.7% ± 9.1% vs. 55.7% ± 13.2% vs. 41.7% ± 17.3%, *p* = 0.387). We determined the survival of the patients grouped according to their MRD level during the third month and investigated whether they had undergone allo-SCT (the survival rates of the patients grouped according to their MRD level on days 14 and 28 after induction therapy initiation were also analyzed, and the outcomes are listed in Additional file 1: Data S2, Fig. S2). The 3-year OS and 3-year DFS of the patients who underwent allo-SCT were much better than those of the patients who did not undergo allo-SCT (3-year OS: 77.8% ± 8.9% vs. 6.7% ± 6.4%, *p* < 0.01; 3-year DFS: 67.4% ± 11% vs. 10% ± 9.5%, *p* < 0.01) (Fig. 1B, C). The 3-year OS and 3-year DFS rates of 27 patients who underwent allo-SCT were different when the patients were grouped according to their third month MRD level (nine positive cases vs. 18 negative cases; 3-year OS: 75.8% ± 12.5% vs. 87.5% ± 11.7%, *p* = 0.567; 3-year DFS: 56.7% ± 15.4% vs. 77.8% ± 13.9%, *p* = 0.753). However, there was no identifiable statistical significance because of the small sample size. In the MRD-negative group, the 3-year OS and 3-year DFS rates were better for the patients who underwent allo-SCT, whereas those who did not undergo allo-SCT still had poor survival rates. According to landmark analysis, there was an obvious significant difference between patients (five cases) who did not undergo allo-SCT and those (18 cases) who underwent allo-SCT in terms of 3-year OS and 3-year DFS (3-year OS: 20% ± 17.9% vs. 75.8% ± 12.5%, *p* = 0.034; 3-year DFS: 20% ± 17.9% vs. 56.7% ± 15.4%, *p* = 0.03) (Fig. 1D, E). Four of the five patients who did not undergo allo-SCT died due to disease relapse.

Overall, our data provide evidence that *TP53* aberrations are critical prognostic factors in adult Ph^−^ ALL and highlight the importance of allo-SCT in the management of ALL patients with *TP53* aberrations. A comparison of the overall remission rates (ORRs) and CR rates after one course of induction therapy did not show substantial differences between patients with and without *TP53* aberrations. Along with previous studies showing MRD level and allo-SCT are crucial prognostic factors in adult ALL patients [8–12], we also analyzed the prognostic value of the third month MRD level and allo-SCT in patients with *TP53* aberrations. Our data showed that allo-SCT could improve the OS of patients with *TP53* aberrations, regardless of aberration types. We also found that avoidance of allo-SCT was associated with a worse 3-year OS and DFS in the patients who achieved an early MRD-negative status. This however differs from a recent study from Ribera et al. [13] where omitting allo-HSCT did not hamper the outcomes of high-risk Ph^−^ ALL patients with adequate MRD response. Moreover, our study first showed *TP53* deletion was of as good prognostic value as the better-studied *TP53* mutaion, which can be incorporated into an improved risk stratification system for adult Ph^−^ALL. Based on these findings, we suggest evaluation of both *TP53* deletion and mutation status in adult patients with Ph^−^ALL at diagnosis and recommend that patients with ALL and any type of *TP53* aberrations should consider allo-SCT.

## Supplementary Information

**Additional file 1: Table S1.** The concomitant gene mutations of patients with *TP53* aberrations. **Table S2.** The clinical characteristics in patients with *TP53* aberrations and without *TP53* aberrations. **Table S3.** The COX regression analysis of the Ph^−^ ALL patients, the covariate including *TP53* aberrations, WBC count, age and whether or not they underwent allo-SCT. **Figure S1.** The 3-year OS (A) and 3-year DFS (B) of patients with *TP53* aberrations compared with patients without *TP53* aberrations in the 137 patients who didn’t undergo allo-SCT. The 3-year OS (C) and 3-year DFS (D) of the 4 different groups (*MLL* rearrangement, *E2A/PBX1*, *TP53* aberrations, other-types) in the 137 patients who didn’t undergo allo-SCT. **Figure S2.** The 3-year OS and 3-year DFS of the patients with *TP53* aberrations who underwent allo-SCT vs. the patients who did not undergo allo-SCT grouped according to MRD level on day 14 (A, B) and day 28 (C, D) from the therapy initiation.

## Data Availability

The clinical trial related information was obtained from public databases.
