# Supplementary material for: The clinical characteristics and prognosis in adult Ph negative acute lymphoblastic leukemia with TP53 aberrations
Source: Exp Hematol Oncol. 2022 Apr 8;11:22. doi: 10.1186/s40164-022-00274-1 (PMC8991885; doi:10.1186/s40164-022-00274-1)
Supplement: Supplementary file 1 — Additional file 1: Table S1. The concomitant gene mutations of patients with TP53 aberrations. Table S2. The clinical characteristics in patients with TP53 aberrations and without TP53 aberrations. Table S3. The COX regression analysis of the Ph− ALL patients, the covariate including TP53 aberrations, WBC count, age and whether or not they underwent allo-SCT. Figure S1. The 3-year OS (A) and 3-year DFS (B) of patients with TP53 aberrations compared with patients without TP53 aberrations in the 137 patients who didn’t undergo allo-SCT. The 3-year OS (C) and 3-year DFS (D) of the 4 different groups (MLL rearrangement, E2A/PBX1, TP53 aberrations, other-types) in the 137 patients who didn’t undergo allo-SCT. Figure S2. The 3-year OS and 3-year DFS of the patients with TP53 aberrations who underwent allo-SCT vs. the patients who did not undergo allo-SCT grouped according to MRD level on day 14 (A, B) and day 28 (C, D) from the therapy initiation. [file 40164_2022_274_MOESM1_ESM.docx]

**Additional file 1**

**Data S1.** The 309 cases were divided into two groups, 45 patients with *TP53* aberrations and 264 patients without *TP53* aberrations. The baseline values of the two groups were almost the same, except for white blood cell (WBC) counts (264 patients without *TP53* aberrations had higher WBC counts) (Table S2), and the 3-year overall survival (OS) and 3-year disease-free survival (DFS) showed no significant differences between the two groups (with *TP53* aberrations vs. without *TP53* aberrations: 3-year OS: 49.4% ± 8.6% vs. 55.7% ± 3.5% *p* = 0.381; 3-year DFS: 52.4% ± 8.8% vs. 53% ± 3.5%, *p* = 0.758). However, of the 137 patients who did not undergo allogeneic hematopoietic stem cell transplantation (allo-SCT), patients (*n* = 15) with *TP53* aberrations showed worse 3-year OS and 3-year DFS rates than patients (*n* = 122) without *TP53* aberrations (3-year OS: 6.7% ± 6.4% vs. 77.8% ± 8.9%, *p* < 0.01; 3-year DFS: 9.1% ± 8.7% vs. 52.9% ± 5.2%, *p <* 0.01, Figure S1 A, B). Patients who underwent allo-SCT showed no significant differences in terms of the presence or absence of *TP53* aberrations (data not shown). We conducted multivariate Cox regression analysis, and *TP53* aberrations was found to be an independent prognostic factor after adjusting for age, WBC count, and allo-SCT (Table S3). Moreover, *MLL* and *E2A/PBX1* fusion genes were detected in the 309 patients. Survival analysis was performed for the following four groups: patients with *MLL* rearrangement, those with *E2A/PBX1*, those with *TP53* aberrations, and those with other mutation types. There were no significant differences in the 3-year OS (*p* = 0.603) and 3-year DFS (*p* = 0.788) rates of the four groups. Next, the four group patients were analyzed depending on whether or not they underwent allo-SCT. In the non-allo-SCT group, patients with *TP53* aberrations had 3-year OS and 3-year DFS rates that were similar to those of patients with *MLL* rearrangement (*TP53* aberrations vs. *MLL* rearrangement: 3-year OS, 7.7% ± 7.4% vs. 16.7% ± 14.8%, *p* = 0.571; 3-year DFS, 7.7% ± 7.4% vs. 11.1% ± 10.5%, *p* = 0.471, Figure S1 C, D) and showed worse survival rates compared with patients with other mutation types (*TP53* aberrations vs. other types: 3-year OS, 7.7% ± 7.4% vs. 62.3% ± 5.5%, *p <* 0.01; 3-year DFS, 7.7% ± 7.4% vs. 49.5% ± 5%, *p <* 0.01, Figure S1 C, D). Thus, on the basis of the abovementioned research results, we considered *TP53* aberrations as a poor independent prognostic factor for patients with Ph^−^ ALL.

**Data S2.** We analyzed the 3-year overall survival (OS) and 3-year disease-free survival (DFS) rates of patients with negative measurable residual disease (MRD) results on days 14 and 28 after induction therapy initiation. MRD was detected on day 14 in 42/45 patients using flow cytometry (three of the 42 patients died during induction therapy after day 14), and 13 patients had MRD < 0.01% (negative). MRD-negative and MRD-positive patients had similar 3-year OS and 3-year DFS rates (MRD-negative patients vs. MRD-positive patients: 3-year OS, 54.7% ± 15.4% vs. 47.6% ± 11.3%, *p* = 0.874; 3-year DFS: 46.7% ± 15.4% vs. 54.6% ± 11.5%, *p* = 0.386). Of the 13 MRD-negative patients, five did not undergo allogeneic hematopoietic stem cell transplantation (allo-SCT) and had poorer outcomes (non-allo-SCT patients vs. allo-SCT patients: 3-year OS, 0% vs. 85.7% ± 13.2%, *p* = 0.001; 3-year DFS: 0% vs. 52.5% ± 20.4%, *p* = 0.002, Figure S2 A, B).

Forty patients had detected MRD on day 28, and 21 patients had MRD < 0.01% (negative). MRD-negative and MRD-positive patients had similar 3-year OS and 3-year DFS rates (MRD-negative patients vs. MRD-positive patients: 3-year OS, 58.1% ± 13.4% vs. 51% ± 12.7%, *p* = 0.296; 3-year DFS: 54.4% ± 13.6% vs. 54.2% ± 12%, *p* = 0.536). Of the 21 MRD-negative patients, although no significant differences were observed due to the small sample size, four patients who did not undergo allo-SCT were likely to have poorer prognosis (patients who did not undergo allo-SCT vs. patients who underwent allo-SCT: 3-year OS, 25% ± 21.7% vs. 72.2% ± 13.8%, *p* = 0.118; 3-year DFS: 25% ± 21.7% vs. 52.5% ± 16.7%, *p* = 0.263, Figure S2 C, D).

|  | KRAS | NRAS | PHF6 | KMT2D | NF1 | Notch1 | ASXL1 | IKZF1 | FLT3 | JAK3 | AKT1 | CDKN2A | CREBBP | CXCR4 | EZH2 | FAT1 | FBXW7 | IDH1 | JAK2 | ETV6 |
| --- | --- | --- | --- | --- | --- | --- | --- | --- | --- | --- | --- | --- | --- | --- | --- | --- | --- | --- | --- | --- |
| TP53^mut^ | 2 | 2 | 3 | 1 | 1 | 1 | 1 | 1 | 1 | 2 | 1 | 1 | 1 | 1 | 0 | 0 | 0 | 1 | 0 | 0 |
| TP53^del^ | 1 | 1 | 0 | 1 | 1 | 1 | 1 | 1 | 1 | 0 | 0 | 0 | 0 | 0 | 1 | 0 | 1 | 0 | 1 | 1 |
| M & D | 1 | 1 | 0 | 0 | 0 | 0 | 0 | 0 | 0 | 0 | 0 | 0 | 0 | 0 | 0 | 1 | 0 | 0 | 0 | 0 |
|  | 8.9% | 8.9% | 6.7% | 4.4% | 4.4% | 4.4% | 4.4% | 4.4% | 4.4% | 4.4% | 2.2% | 2.2% | 2.2% | 2.2% | 2.2% | 2.2% | 2.2% | 2.2% | 2.2% | 2.2% |

**Table S1.** The concomitant gene mutations of patients with *TP53* aberrations.

Note: mut: mutations; del: deletions; M&D: mutations and deletions.

|  | Gender |  | Age | WBC | Cytogenetic risk | |
| --- | --- | --- | --- | --- | --- | --- |
|  | M | F |  |  | LR | HR |
| TP53 aberrations  (N=45) | 25 | 20 | 27 | 5.46 | 14 | 31 |
|  | (55.6) | (44.4%) | (14-67) | (0.73-245) | (31.1%) | (68.9%) |
| Other  (N=265) | 158 | 107 | 26 | 12.8 | 79 | 185 |
|  | (59.8%) | (40.5%) | (13-69) | (0.59-504) | (29.9%) | (70.1%) |
| P value | 0.624 | | 0.585 | 0.013* | 0.862 | |

**Table S2.** The clinical characteristics in patients with *TP53* aberrations and without *TP53* aberrations.

Note: LR: low risk; HR: high risk

|  | WBC | Age | TP53 aberrations | Allo-SCT |
| --- | --- | --- | --- | --- |
| P value | 0.123 | 0.001 | 0.049 | ＜0.01 |
| HR | 1.002 | 1.021 | 1.66 | 0.407 |
| 95% CI | (1-1.004) | (1.009-1.034) | (1.001-2.753) | (0.276-0.601) |

**Table S3.** The COX regression analysis of the Ph^-^ ALL patients, the covariate including *TP53* aberrations, WBC count, age and whether or not they underwent allo-SCT.

1. **B.**


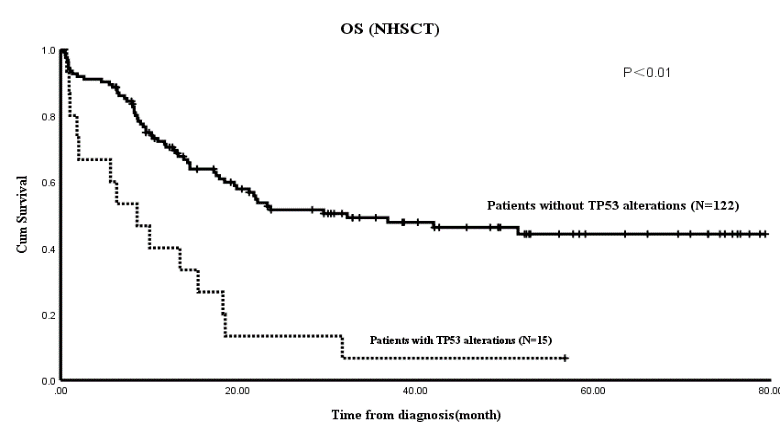

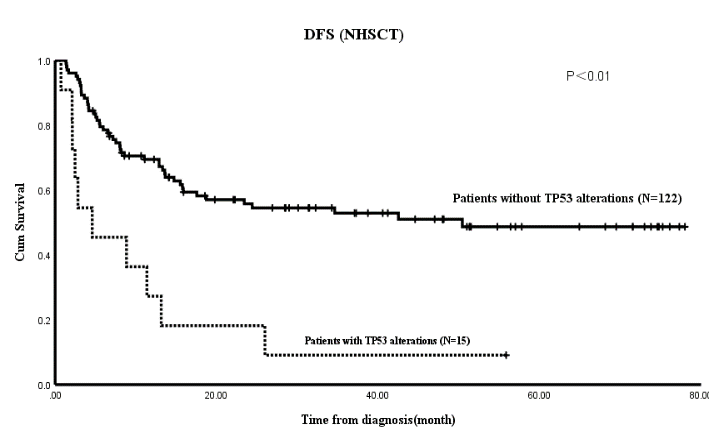


1. **D.**


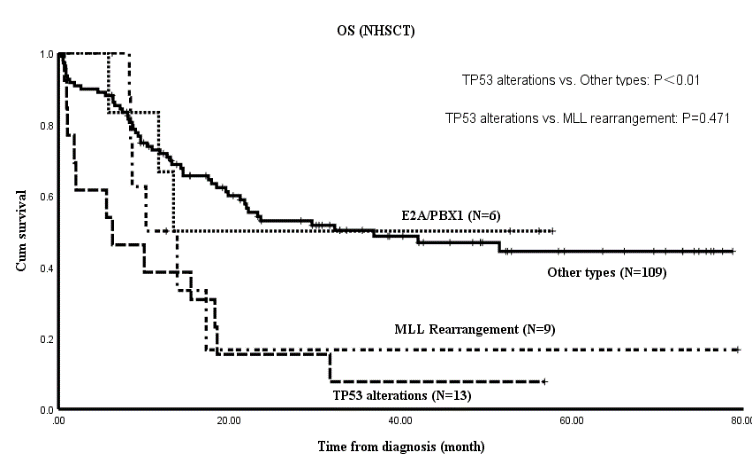

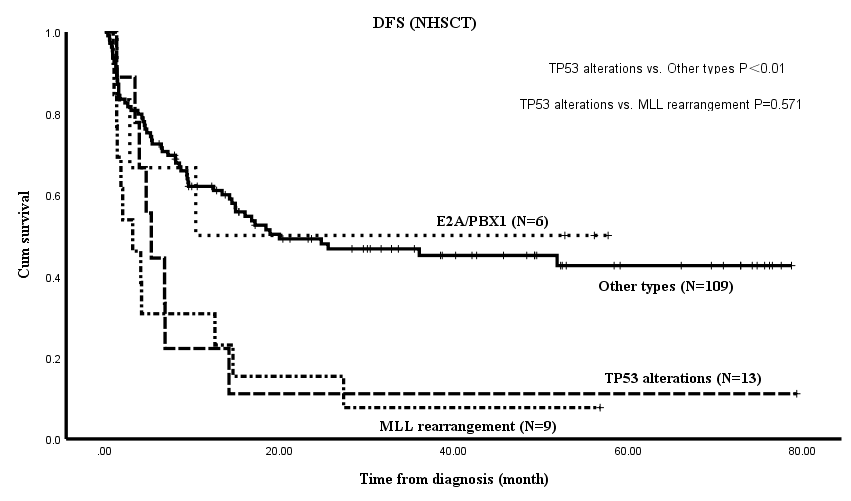


**Figure S1.** The 3-year OS (A) and 3-year DFS (B) of patients with *TP53* aberrations compared with patients without *TP53* aberrations in the 137 patients who didn’t undergo allo-SCT. The 3-year OS (C) and 3-year DFS (D) of the 4 different groups (*MLL* rearrangement, *E2A/PBX1*, *TP53* aberrations, other-types) in the 137 patients who didn’t undergo allo-SCT.

1. B.


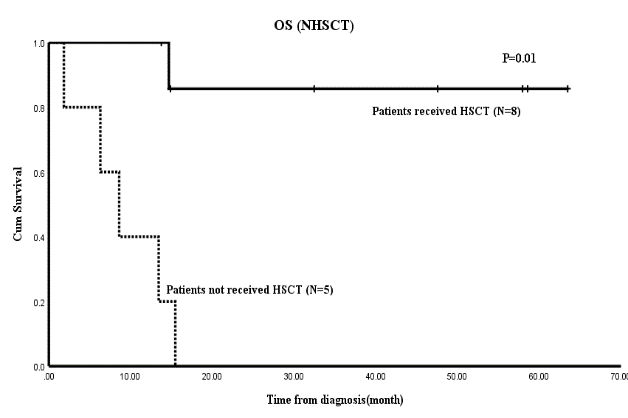

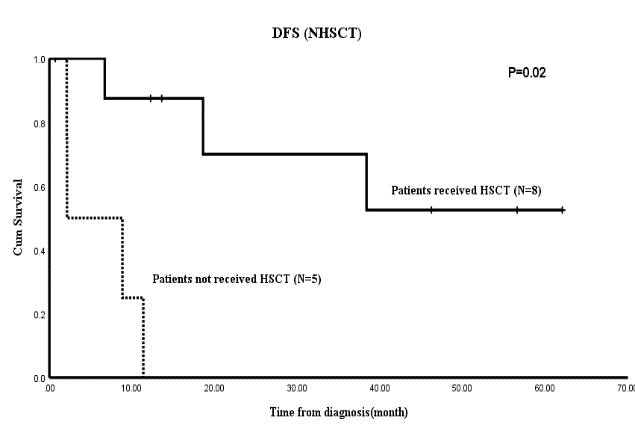


C. D.


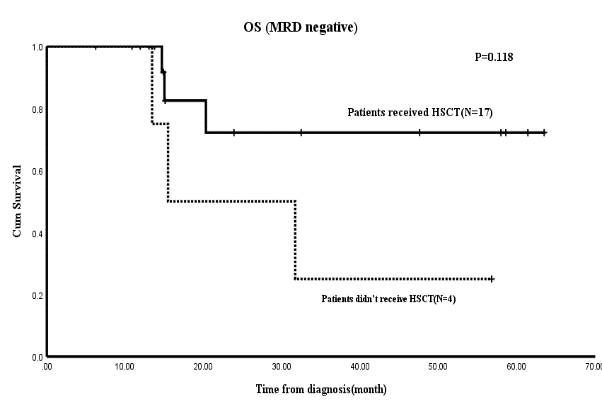

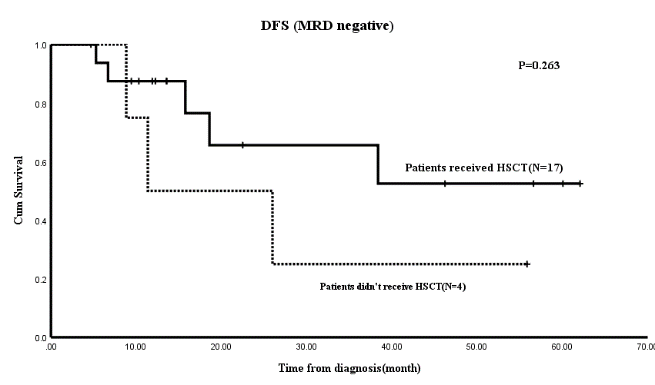


Figure S2: The 3-year OS and 3-year DFS of the patients with *TP53* aberrations who underwent allo-SCT vs. the patients who did not undergo allo-SCT grouped according to MRD level on day 14 (A, B) and day 28 (C, D) from the therapy initiation.
